# Uninterrupted DOACs Approach for Catheter Ablation of Atrial Fibrillation: Do DOACs Levels Matter?

**DOI:** 10.3389/fcvm.2022.864899

**Published:** 2022-03-29

**Authors:** Michael Hardy, Jonathan Douxfils, Anne-Sophie Dincq, Anne-Laure Sennesael, Olivier Xhaet, Francois Mullier, Sarah Lessire

**Affiliations:** ^1^Université catholique de Louvain, Hematology Laboratory, Namur Thrombosis and Hemostasis Center, Namur Research Institute for Life Sciences, Centre Hospitalier Universitaire UCL Namur, Namur, Belgium; ^2^Université catholique de Louvain, Department of Anesthesiology, Namur Thrombosis and Hemostasis Center, Namur Research Institute for Life Sciences, Centre Hospitalier Universitaire UCL Namur, Namur, Belgium; ^3^Department of Pharmacy, Namur Thrombosis and Hemostasis Center, Namur Research Institute for LIfe Sciences, University of Namur, Namur, Belgium; ^4^QUALIblood s.a., Namur, Belgium; ^5^Université catholique de Louvain, Pharmacy Department, Namur Thrombosis and Hemostasis Center, Namur Research Institute for Life Sciences, Centre Hospitalier Universitaire UCL Namur, Namur, Belgium; ^6^Université catholique de Louvain, Department of Cardiology, Namur Thrombosis and Hemostasis Center, Centre Hospitalier Universitaire UCL Namur, Namur, Belgium

**Keywords:** atrial fibrillation, catheter ablation, direct oral anticoagulant, unfractionated heparin, activated clotting time

## Abstract

Most patients present for catheter ablation of atrial fibrillation (CAAF) with residual or full effect of vitamin K antagonists (VKAs) or direct oral anticoagulants (DOACs). In daily practice, it has been observed that the activated clotting time (ACT) was actually poorly sensitive to the effect of DOACs and that patients on DOACs required more unfractionated heparin (UFH) to achieve the ACT target of 300 s during the procedure, leading some authors to worry about potential overdosing. Conversely, we hypothesize that these higher doses of UFH are necessary to achieve adequate hemostasis during CAAF regardless of the residual effect of DOACs. During CAAF, thrombosis is promoted mainly by the presence of thrombogenic sheaths and catheters in the bloodstream. Preclinical data suggest that only high doses of DOACs are able to mitigate catheter-induced thrombin generation, whereas low dose UFH already do so. In addition, the effect of UFH seems to be lower in patients on DOACs, compared to patients on VKAs, explaining part of the differences observed in heparin requirements. Clinical studies could not identify increased bleeding risk in patients on DOACs compared to those on VKAs despite similar efficacy during CAAF procedures. Moreover, targeting a lower ACT was associated with an increased periprocedural thrombotic risk for both DOAC and VKA patients. Therefore, the low sensitivity of the ACT to the residual effect of DOACs should not be a major concern in its use in the interventional cardiology laboratory.

## Introduction

Atrial fibrillation (AF) is associated with a significant thrombotic risk, requiring long-term anticoagulation in patients with intermediate or high thrombotic risk (1–4). Nowadays, vitamin K antagonists (VKAs) and direct oral anticoagulants (DOACs) are the main anticoagulants for stroke prevention in non-valvular AF (1–3). Over the years, catheter ablation of atrial fibrillation (CAAF) has become a first- or second-line treatment for symptomatic AF (5). However, the procedure is associated with a thrombotic risk and requires the administration of high-dose unfractionated heparin (UFH; between 50 and 120 units per kg just before or immediately after transseptal puncture), exposing patients to a risk of bleeding (5); reported incidences of bleeding (e.g., groin bleeding/hematoma, cardiac tamponade) and embolic (e.g., transient ischemic attacks, strokes) complications during hospitalization being ~1.9 and 0.2%, respectively (6). Before ablation, most patients receive anticoagulation for at least 3 weeks to reduce the thromboembolic risk associated with CAAF (5).

Historically, VKAs therapy was interrupted and “bridged” with low molecular weight heparins (LMWH) before and after CAAF. In 2014, the COMPARE randomized trial identified a lower rate of periprocedural stroke and minor bleeding when warfarin was continued with an INR in the therapeutic range (i.e., between 2.0 and 3.0) throughout the periprocedural period, compared with discontinuation with LMWH bridging (7). With the introduction of DOACs in non-valvular AF patients, due to the concern of potentially major bleeding and the lack of a convenient reversal agent, DOACs were discontinued in the preprocedural period. Since then, several randomized trials have compared CAAF with uninterrupted DOAC vs. uninterrupted VKA approaches and found no significant differences between the two groups in terms of thrombotic and bleeding complications (8–15). This approach is now recommended over discontinuation and bridging (5). An acceptable alternative to avoid high DOAC peak plasma concentrations during CAAF is to skip one or two DOAC doses before the procedure (16). However, clinical trials that analyzed uninterrupted or minimally interrupted approaches for DOACs were all underpowered.

During the procedure, repeated measurements of the activated clotting time (ACT) each 10–15 min intervals until therapeutic anticoagulation and then at 15–30 min intervals are recommended to guide UFH administration (5). The test, which consists in measuring the time to clot formation in whole blood after complete activation of the contact pathway (e.g., celite or kaolin), is poorly sensitive to DOACs, especially to direct factor Xa inhibitors, even at high concentrations corresponding to the peak effect (17). An ACT maintained above 300 s is recommended during CAAF on VKA therapy based on studies visualizing a lower incidence of thrombi in left heart chambers and according to observational studies (5, 18–20). This threshold is applied similarly for CAAF on DOAC therapy despite a lower level of evidence. However, higher UFH doses are required to achieve the ACT goal of 300 s when DOACs are on board, compared to VKAs (8, 15, 18, 21–27), which corresponds to a potential overdosage according to some authors (21, 28). Conversely, we hypothesize that these higher doses of UFH are necessary to achieve adequate hemostasis during the procedure and that considering the residual effect of DOACs would not be as important as expected. The following paper will get some insights into the mechanisms of thrombosis during CAAF and the possibilities to manage them.

## Discussion

### Mechanisms of Thrombosis During CAAF by Thermocoagulation

**Figure 1:**
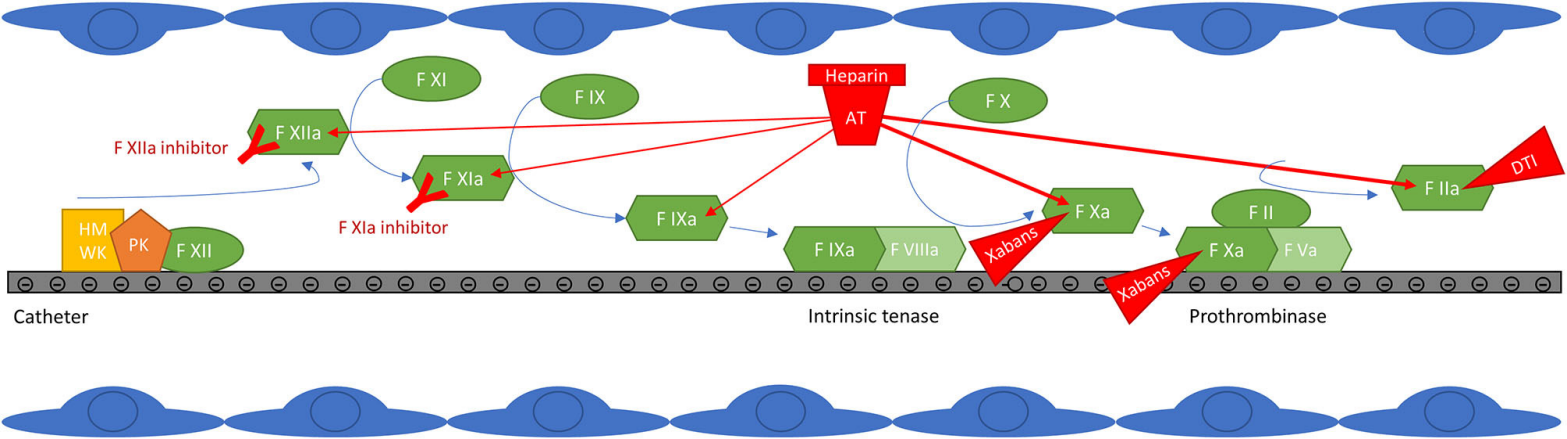
Contact pathway activation at the surface of catheters and targets of anticoagulant drugs. Factor XIIa auto-activates on contact with negatively charged surfaces. High molecular weight kininogen (HMWK) is also adsorbed and serves as a cofactor for FXIIa auto-activation. FXIIa then activates FXI to initiate thrombin generation, but also prekallikrein (PK) to kallikrein, resulting in further FXIIa generation. FXIa activates FIX, which forms the intrinsic tenase with FVIIIa. Intrinsic tenase and prothrombinase complexes form on negatively charged surfaces, classically on the surface of activated platelets, but possibly also on the surface of catheters. The thrombin generated then amplifies the reaction by promoting the activation of factors XI, VIII and V, but it is also a potent platelet activator. In addition to the generation of thrombin initiated by FXIIa, other thrombotic mechanisms take place on the surface of catheters (not shown in the figure): the adsorption of proteins such as fibrinogen, which promotes the adhesion and activation of platelets and leukocytes, potentially increasing thrombin generation and thrombogenesis (47–51). FXIIa, kallikrein and thrombin also activate the complement system which promotes platelet activation and thrombin generation (53–57). The effect of anticoagulant drugs is also depicted: unfractionated heparin (UFH) potentiates the inhibitory effect of antithrombin (AT) mainly on free activated factors X and II, but also to a lesser extent on free activated factors IX, XI and XII (86); clot-bound FXa and FIIa, and FXa within the prothrombinase complex, are less accessible to inhibition by AT (87–89). Direct oral anticoagulants (direct F Xa inhibitors (xabans) and the direct thrombin inhibitor (DTI) dabigatran) directly inhibit activated factor II or X, free, within the prothrombinase complex and clot-bound (89–92). Contact pathway inhibitors specifically target factor XI or XII; truncated antibodies against FXIa or FXIIa are shown in the figure, but this category also includes small inhibiting molecules, antisense oligonucleotides and small interfering RNAs (82).

In addition to possible pre-existing thrombi that may be dislodged by the catheters or by fluctuations in heart rhythm during the procedure, several mechanisms have been advocated to explain thrombi formation during CAAF (29, 30). First, direct endothelial damage may result from the passage of sheaths and catheters from the femoral vein to the left atrium and from thermal injuries during the ablation procedure; radiofrequency ablation could be more thrombogenic than cryoablation by this mean (31). Second, the contact pathway is activated on the surface of foreign materiel (sheaths, catheters) in the bloodstream (Figure 1) and by cell debris such as DNA or polyphosphates released during thermoablation. Occasionally, emboli may also arise from coagulum or char formation on the ablation electrode, which can be limited by proper technique and does not seem to depend on hemostasis and anticoagulation (32). Of those thrombus/coagulum sources, those formed in the left atrium are particularly dangerous because they are more likely to embolize into the systemic circulation, whereas emboli formed in peripheral veins or right chambers can only embolize into the systemic circulation through an interatrial communication.

During CAAF, intracardiac ultrasound can be used to directly visualize thrombi formed in the cardiac chambers. Small observational studies identified that thrombi form primarily on transseptal sheaths or on mapping catheters (19, 20, 33–36). Less frequently, thrombi are also seen in the left atrium, pulmonary vein or left atrial appendix; however, as some of these thrombi can be extracted by strong suction through the sheath during its removal, some authors emphasized that those thrombi could have been initially related to the sheath or the catheter itself (35). It should be noted that no thrombi are generally observed on the ablation lesion itself during the procedure and radiofrequency ablation is not associated with an increase in *in vivo* thrombin generation markers (such as thrombin-antithrombin complexes), compared with the mapping phase or with single electrophysiological studies (33, 37–39). Taken together, these data suggest that sheaths/catheters may be the main source of intracardiac thrombus formation during CAAF.

The thrombogenicity of catheters has been studied in preclinical models (40–46). Yau et al. demonstrated *in vitro* that clot forms three times faster in the presence of catheters than in their absence (41). They identified that coagulation was activated on the surface of catheters via the contact pathway, as the procoagulant effect of the catheters was reduced or reversed by corn trypsin inhibitor, in plasma deficient in factor XI or XII, or in rabbits treated with antisense oligonucleotide for factor XII or XI (41, 42, 45). Another research group demonstrated that *Ixodes ricinus Contact Pathway Inhibitor (Ir-CPI)* also has the potential to reduce the procoagulant effect of catheters *in vitro* (46). Interestingly, they showed that catheters were still able to induce a procoagulant effect in factor XII deficient plasma which could be abolished by the presence of Ir-CPI, suggesting that factor XII is not the only coagulation factor implicated in the activation of the coagulation cascade by catheters (46). As Ir-CPI is a dual inhibitor of both factor XII and factor XI, it is suggested that factor XI is also involved in the thrombogenesis mechanism of catheter-induced thrombosis.

As catheters are the primary site of thrombus formation during CAAF and as these catheters generate thrombin via the contact pathway, contact pathway inhibition may represent the primary target of anticoagulation during CAAF. Although tissue factor (TF) pathway could also contribute to thrombogenesis during the procedure (endothelial lesions by the passage of the sheaths and following cellular destruction during transseptal puncture or the application of thermal energy), UFH at concentrations required to block contact activation would also provide protection on TF-initiated thrombin generation.

Besides direct activation of coagulation upon contact with negatively charged surfaces, other mechanisms can contribute to thrombin generation during CAAF. First, proteins such as fibrinogen are adsorbed to the catheter surface, which promotes platelet adhesion and activation (47–49). Leukocytes can also adhere to adsorbed fibrinogen and platelets (50, 51); leukocytes may then degranulate and promote inflammation. Neutrophils extracellular traps can also activate the coagulation contact pathway and contribute to thrombin generation (52). In addition, the complement system may be activated by FXIIa, kallikrein and thrombin, which may then promote thrombosis through platelet activation and direct thrombin generation (53–57). However, it is less clear to what extent these mechanisms would contribute to thrombogenesis and how clinicians might manage them. Finally, direct measurement of haemostasis proteins/biomarkers in cardiac chambers could be more sensitive and help to understand pathogenesis more precisely (58, 59).

### Pharmacological Prevention of Contact Phase Activation

Among the conventional anticoagulants, heparins are preferred for contact inhibition in the acute setting [e.g., CAAF, extracorporeal circuits, mechanical heart valves (MHV)] (Figure 1). Previous work identified that inhibition of catheter-induced thrombin generation was more effective with UFH than with LMWH and poorly effective with fondaparinux, which was also ineffective at blocking FXIIa- and FXIa-initiated thrombin generation (41). Similar results were also observed in a rabbit model of catheter thrombosis (41). This could be due to the greater anti-IIa activity of UFH, compared to LMWH or fondaparinux, or to its upstream effect on free FIXa (60).

Whereas, UFH strongly inhibits contact-initiated thrombin generation, the ability of DOACs to do so may be much less. For example, only dabigatran concentrations of 200 ng/mL and above were able to attenuate *in vitro* polyurethane catheter-induced thrombin generation, whereas UFH concentrations as low as 0.02 IU/mL could already do so (43). No data are available regarding the ability of direct anti-Xa to mitigate catheter-induced thrombin generation. However, preclinical studies are available in other contact pathway activation models such as mechanical heart valves (MHV). As with catheters models, dabigatran, but also apixaban and rivaroxaban had limited ability to suppress MHV-induced thrombin generation at concentrations consistent with those observed in therapeutically anticoagulated patients (61, 62). Therefore, it is questionable whether full consideration of residual DOACs levels is relevant for thrombosis prevention during CAAF.

Another important aspect to consider is the pharmacodynamic interaction between UFH and oral anticoagulants. Unfortunately, few data are available regarding this topic. A pharmacokinetic-pharmacodynamic study performed in CAAF patients identified that the response to intravenous UFH was similar between patients on dabigatran and patients without baseline anticoagulation (same ACT increase for a given UFH bolus), but was enhanced in patients on VKAs (increased ACT increase for a given UFH bolus) (63). However, dabigatran was often skipped for one dose before the procedure, probably resulting in low plasma concentrations, which limits the findings of the study. Using thrombin generation, other authors identified a reduced response to the *in vitro* addition of 0.1 IU/mL UFH to the plasma of patients on DOACs (both at Cpeak and Ctrough), compared with the plasma of patients on VKA or healthy volunteers. The response to UFH was greater for samples with dabigatran than for samples with direct anti-Xa inhibitors (64). Finally, Yau et al. identified a synergistic effect on delaying the time to catheter occlusion in rabbits when low-dose dabigatran and UFH were administered concomitantly (43).

### Monitoring of UFH During CAAF—The Activated Clotting Time

During CAAF, UFH administration is guided using the ACT. A variety of devices and cartridges are available, differing in the activator used (e.g., celite, kaolin, glass beads or a combination of these) and the method of measurement (e.g., rotation of a tube, a plunger or movement through capillaries) (17). Systematic differences exist between available ACT devices [which may be more than 100 s in heparinized patients (65–68)], which cannot be used interchangeably (65–71). However, in clinical guidelines, fixed ACT targets (i.e., 300 s) are proposed without differentiating devices (5), which adds variability in the level of anticoagulation achieved from center to center. In addition, various preanalytical variables may influence the ACT, such as blood collection technique (e.g., site of blood collection, amount of blood discarded before sampling, velocity of aspiration during sampling) and processing (e.g., time-interval between collection and analysis, agitation of the sample, prewarming of the reagent) (72, 73). As a result, this could also lead to huge variations in UFH dose administered.

Although the excellent correlation between ACT and UFH concentrations with *in vitro* spiking of whole blood (74, 75), the association between ACT and *ex vivo* heparin levels assessed with an anti-Xa assay is poor, especially at high UFH concentrations such as those used during CAAF (i.e., 1–2 IU/mL) (76). Unlike UFH, the ACT shows poor sensitivity to DOACs *in vitro*, especially to direct factor Xa inhibitors (inability to achieve ACTs >200 s even at supratherapeutic concentrations), whereas its sensitivity to dabigatran, the only direct factor IIa inhibitor, is better (77). When using samples from patients on DOAC, the correlation with direct factor Xa inhibitors levels is even worse (28). As a result, and because of uninterrupted preoperative anticoagulation attitudes, some patients may present in the interventional cardiology laboratory with therapeutic DOACs blood levels with only small ACT prolongations, especially for direct factor Xa inhibitors (78, 79).

### Outcomes in Clinical Studies

Some authors suggested that the higher doses of UFH administered to patients on DOACs, compared with patients on VKAs, could be detrimental by adding to the residual effect of uninterrupted DOACs, putting patients at increased bleeding risk (21, 28). However, meta-analyses of randomized trials and observational studies comparing uninterrupted VKA and DOAC treatment approaches are reassuring, identifying no increase in bleeding risk with DOACs compared with VKAs (80, 81); dabigatran was even safer than VKAs in the RE-CIRCUIT randomized trial [absolute risk difference −5.3% (95% confidence interval: −8.4 to −2.2%), *p* < 0.001] (10). Furthermore, these increased doses of UFH appear to be necessary to prevent thrombosis during CAAF. Indeed, another meta-analysis identified that, similarly to the uninterrupted VKAs approach, achieving an ACT >300 s for patients on DOAC therapy was associated with a reduced risk of thromboembolic events, compared with an ACT target of <300 s (18). Overall, this suggests that the lack of integration of DOACs levels by the ACT and the hassle of administrating higher UFH doses with the uninterrupted DOAC approach are not worrying in terms of clinical endpoints. Worse, aiming for lower ACTs for fear of overanticoagulation could be deleterious by increasing thrombotic risk.

### Future Directions

Due to the expected predominant role of the contact pathway in procedural thrombosis, contact pathway inhibitors are attractive for anticoagulation during CAAF. Contact pathway inhibitors are pharmacologic agents targeting specifically factor XII or XI using truncated antibodies, small inhibiting molecules, antisense oligonucleotides (ASO) or small interfering RNAs (82). These molecules are able to profoundly block contact activation with no effect on hemostasis initiated by TF exposure. At present, these molecules have been used successfully in preclinical models of extracorporeal life support (83, 84), and were associated with excellent thrombosis prevention with minimal bleeding risk (sometimes lower than UFH). In human, FXI ASO were more effective than LMWH to prevent venous thrombosis after total knee arthroplasty with a lower incidence of clinically relevant bleeding (85). However, the utilization of these molecules during CAAF should be done with caution as the contribution of TF-induced thrombin generation to thrombosis during the procedure remains unresolved. Whether this mechanism does significantly contribute to thrombosis, and whether residual VKAs or DOACs concentrations in the context of uninterrupted approaches would be enough to counter this specific risk would deserve to be carefully studied.

## Conclusion

Although the activated clotting time is poorly sensitive to the effect of direct factor Xa inhibitors, the latter may not be very effective in mitigating catheter-induced thrombin generation, at least at concentrations encountered in the interventional cardiology laboratory. Furthermore, the higher UFH doses required to achieve the ACT target of 300 s in patients on uninterrupted DOAC therapy, compared with those required in an uninterrupted VKA approach, do not appear to dangerously compromise the hemostatic competence of those patients, as evidenced by available randomized controlled trials and meta-analysis. Although the reliability of the ACT for assessing overall coagulation in the presence of high-dose heparin may still be questioned, its low sensitivity to the residual effect of direct factor Xa inhibitors is not a major concern in its use in the interventional cardiology laboratory.

## Author Contributions

MH, FM, and SL wrote the first draft of the manuscript. All authors contributed to manuscript writing, revision, read, and approved the submitted version.

## Conflict of Interest

JD is the CEO and founder of QUALIblood s.a., a contract research organization manufacturing the DP-Filter, is a coinventor of the DP-Filter (patent application number: PCT/ET2019/052903) and reports personal fees from Daiichi-Sankyo, Mithra Pharmaceuticals, Stago, Roche and Roche Diagnostics outside the submitted work. FM reports institutional fees from Stago, Werfen, Nodia, Roche Sysmex and Bayer. He also reports speaker fees from Boehringer Ingelheim, Bayer Healthcare, Bristol-Myers Squibb, Pfizer, Stago, Sysmex and Aspen all outside the submitted work. The remaining authors declare that the research was conducted in the absence of any commercial or financial relationships that could be construed as a potential conflict of interest.

## Publisher's Note

All claims expressed in this article are solely those of the authors and do not necessarily represent those of their affiliated organizations, or those of the publisher, the editors and the reviewers. Any product that may be evaluated in this article, or claim that may be made by its manufacturer, is not guaranteed or endorsed by the publisher.
